# Resilience to orthostasis and haemorrhage: A pilot study of common genetic and conditioning mechanisms

**DOI:** 10.1038/srep10703

**Published:** 2015-05-29

**Authors:** Dmitry M. Davydov, Renad I. Zhdanov, Vladimir G. Dvoenosov, Olga A. Kravtsova, Elena N. Voronina, Maxim L. Filipenko

**Affiliations:** 1 Sholokhov Moscow State University for the Humanities, the Russian Institute for Advanced Study and Institute of Neurosciences and Cognitive Research, Verkhnyaya Radishevskaya 16-18, Moscow, 109240; 2 Laboratory of Neuroimmunopathology, Institute of General Pathology and Pathophysiology, Russian Academy of Medical Sciences, 8 Baltiyskaia ul., Moscow, 125315, Russia; 3 Institute of Fundamental Medicine and Biology, Kazan (Volga region) Federal University, 18 Kremlin ul., Kazan, 420008, Russia; 4 Pharmacogenomics laboratory, Institute of Chemical Biology and Fundamental Medicine, Siberian Branch, Russian Academy of Sciences, Novosibirsk, 630090, Russia

## Abstract

A major challenge presently is not only to identify the genetic polymorphisms increasing risk to diseases, but to also find out factors and mechanisms, which can counteract a risk genotype by developing a resilient phenotype. The objective of this study was to examine acquired and innate vagal mechanisms that protect against physical challenges and haemorrhages in 19 athletes and 61 non-athletes. These include examining change in heart rate variability (HF-HRV; an indicator of vagus activity) in response to orthostatic challenge, platelet count (PLT), mean platelet volume (MPV), and single-nucleotide polymorphisms in genes that encode several coagulation factors, PAI-1, and MTHFR. Individual differences in PLT and MPV were significant predictors, with opposite effects, of the profiles of the HF-HRV changes in response to orthostasis. Regular physical training of athletes indirectly (through MPV) modifies the genetic predisposing effects of some haemostatic factors (PAI-1 and MTHFR) on vagal tone and reactivity. Individual differences in vagal tone were also associated with relationships between *Factor 12 C46T* and *Factor 11 C22771T* genes polymorphisms. This study showed that genetic predispositions for coagulation are modifiable. Its potential significance is promoting advanced protection against haemorrhages in a variety of traumas and injuries, especially in individuals with coagulation deficits.

‘Resilience’ and ‘adaptation’ are two major concepts used to explain the capacity of a living organism to flourish in the face of adversity and to effectively maintain its health and well-being^1^,^2^,^3^. Resilience contributes to survival and mental and physical fitness by maintaining biological or psychosocial stability in response to potential risks. In contrast, adaptation contributes to survival and fitness by maintaining biological or psychosocial flexibility in response to imposed conditions. Individuals may be genetically or epigenetically predisposed to more flexible adaptation or to more rigid resilience. These mechanisms should be well-balanced in organisms for their final result achieves a good health.

For example, the evaluation of autonomic regulation of the heart rate, which is indicated by its variability changes in response to an orthostatic challenge, has been suggested to predict an individual’s capacity for adaptive responses of the sympathetic nervous system (SNS)^4^,^5^,^6^,^7^,^8^,^9^,^10^,^11^. However, withdrawal of these adaptive changes or recovery after an orthostatic challenge should be considered an individual’s capacity for resilience or recovery status of the parasympathetic nervous system (PNS)^8^,^12^,^13^,^14^,^15^,^16^,^17^. At the same time the impaired adaptive ability to maintain blood pressure with sympathetic control in response to an orthostatic challenge is associated with a reduced ability of the vagus to regulate recovery of the heart at rest^18^. Thus an increase in baseline vagus or PNS activity increases the SNS capacity to withstand an orthostatic challenge, i.e., higher resilience capacity allows the organism to be more flexible or adaptive in responses to challenges without health impairments.

PNS activity, as a physiological resilience factor against exacerbated sympathetic arousal, is suggested to exert a generalized tonic effect across different physiological (e.g., cardiovascular, metabolic, immune, haemostatic) systems for survival and to provide permanent protection of the organism from impairments associated with physiological over-arousal^19^. For example, electrical stimulation of the vagus nerve was observed to attenuate the mechanical and metabolic responses of the heart to beta-adrenergic stress by reducing cardiac activity and oxygen, glucose and lactate consumption^20^. It also lessens peripheral haemorrhaging after severe (hemorrhagic shock) and mild (soft tissue injury) hemorrhage by improving coagulation, reducing bleeding time and total blood loss^21^,^22^. In the case of hemorrhage, the protective effect is associated with improved clot formation kinetics, enhanced clot firmness, and increased coagulation factor activity (thrombin-antithrombin complex formation). An enhanced tonic activation of postsynaptic serotonin receptors in response to vagus nerve activation may be the mechanism promoting clot formation at the site of vascular injury^23^,^24^. In both cases the loss of vagus regulation should essentially cause physiological over-arousal, that exacerbates damage to the organism. Thus, individuals, whose parasympathetic recovery in the cardiovascular (CV) system is diminished or incomplete after a high arousal challenge may also show a coupled bias in haemostatic variables, indicating a predisposition to a lower coagulatory state.

Previously relationships between CV activity and haemostatic factors have been mostly estimated with respect to only blood pressure changes, which do not correlate with heart rate variability changes as an indicator of parasympathetic activity^25^. Other studies have investigated the relationship between CV activity and haemostatic variables in patients. For example, a recent study observed a lower resting heart rate variability in patients with acute coronary syndrome who also showed increased levels of von Willebrand factor, soluble P-selectin and D-dimer compared to healthy subjects, but did not detect correlations between different indexes of heart rate variability and these thrombotic/haemostatic markers^26^. Other studies have found that the power of a high frequency component of heart rate variability (HF-HRV) obtained during 24-hour monitoring was inversely associated with both a composite prothrombotic index and individual levels of haemostatic factors, including von Willebrand factor antigen (VWF:Ag), activated clotting factor VII, fibrinogen, plasminogen activator inhibitor-1 (PAI-1), and mean platelet volume (MPV)^27^,^28^. In healthy subjects VWF:Ag was inversely correlated with the platelet count (PLT) and positively correlated with MPV^29^. Platelet activation was found to cause bradycardia and hypotension by a general increase in vagal tone and a decrease in sympathetic tone^30^. This is caused by serotonin released from platelets. Diminished serotonergic activity in carriers of short/short genotype of the serotonin transporter-linked polymorphic region causes diminished cardiac vagal activity under basal conditions and blunted autonomic reactivity as assessed by heart rate variability (HRV)^31^. Serotonin activates megakaryocyte apoptosis, platelet activation and potentiates their overall procoagulant activity^24^,^32^. Thus the serotonergic system may be responsible for transmitting a general physiological resilience effect from the PNS to specific systems for withstanding different challenges (Fig. 1a).

The present study was conducted to confirm the main assumption about vagus activity (phenotyped by HF-HRV) as a protective (resilience) factor in response to regular everyday actions such as physical activity (e.g., orthostatic challenge or change in posture), which should also predict a vulnerability to haemostasis or haemorrhages (phenotyped by the platelet index [PLT and MPV])^33^,^34^,^35^,^36^. Another objective of the study was to observe whether the protective effects of vagal activity could be related to innate mechanisms (an innate resilience determined by single-nucleotide polymorphisms (SNP) in genes of coagulation factors II, V, XI, XII [*F2, F5, F11, F12*], plasminogen activator inhibitor-1 (*PAI-1*), glycoprotein 3a [*GP3a*], methylenetetrahydrofolate reductase [*MTHFR*]) or could be acquired due to conditioning mechanisms (an acquired resilience determined by aerobic fitness) that differ between athletes (higher fit adults) and nonathletes (lower fit adults). Elucidation of the mechanisms of vagus action could reveal novel therapeutic and pre-conditioning approaches for a variety of combined stress-, trauma-, and haemorrhage-related applications.

The research was planned as pilot study and therefore was limited to the genotyping of mutations in haemostatic factor genes that control either (i) extrinsic and intrinsic clotting paths conferring an inherited thrombophilia tendency in normal and patho-physiological conditions (FII, FV, FXI, FXII, GP3a, and MTHFR) or (ii) fibrinolytic activity conferring an inherited tendency for greater blood loss during injuries (PAI-1). Haemostatic phenotype was restricted to two platelet indices: first (MPV) that previously showed evidence of significant relationship with selected physiological marker of vagus activity (i.e., HRV)^28^ and second (PLT) that was not found to be studied yet with respect to HRV. As shown previously both haemostatic indices have significant relationships with some other important haemostatic factors (e.g., alpha(2)-plasmin inhibitor-plasmin complex, interleukin-6, thrombin-antithrombin III complex and VWF:Ag)^29^,^37^

## Results

### Sample characteristics

No deviation from the Hardy–Weinberg equilibrium was observed in the athlete and non-athlete samples either separately or combined. Demographic and cardiovascular characteristics and genotype frequencies of athlete and non-athlete samples are shown in Tables 1 and 2. Non-adjusted scores of interbeat intervals (IBI) and the power of a high frequency component of the HRV (HF-HRV) were significantly higher in athletes compared to non-athletes during the baseline and recovery periods, but athletes had significantly lower scores of MPV (Table 1). The distribution of women and men and Factor 5 genotype variants were significantly different between athletes and non-athletes (Tables 1 and 2). All other presented results are controlled for gender and age.

### Relationships between haemostatic and cardiovascular variables

#### Platelet number

Significant Posture_*_PLT interactions were obtained for vagus tone (lnHF-HRV) and vagus reactivity (reHF-HRV) measures (Table 3). Figure 2A,B present this platelet number effect on the profile of between-posture HF-HRV changes with 242 as the cut-off point for this haemostatic factor (Pillai’s Traces = 0.25 and 0.21, Fs(2) = 6.46 and 5.04, ps = .004 and .011 for absolute and relative scores, respectively). Individuals with a lower platelet number showed a flatter profile of HF-HRV changes in response to posture changes compared to those with a higher platelet number. Post-hoc analysis found that parameter estimates of reHF-HRV during the lying1 (baseline) position were significantly lower, but during standing were higher in individuals with a lower platelet number compared to those with a higher platelet number (Bs[SE] = −793.3[307.1] and 777.5[246.2], t[p] = −2.58[.014] and 3.16[.003], η^2^ = .15 and .20, respectively). Bootstrap procedure confirmed the observed PLT effects with 95% confidence (data not shown).

#### Mean platelet volume

Significant Posture_*_MPV interaction effect on lnHF-HRV was obtained (Table 3). Figure 2C presents the mean platelet volume effect on a profile of between-posture HF-HRV changes with 8.6 as the cut-off point of this haemostatic factor (Pillai’s Trace = 0.24, F(2) = 5.84, p = .006). Individuals with a higher mean platelet volume showed a flatter profile of HF-HRV changes in response to posture changes compared to those with a lower mean platelet volume. Post-hoc analysis found that parameter estimates of lnHF-HRV during both the lying1 (baseline) and lying2 (recovery) positions were significantly lower in individuals with a higher mean platelet volume compared to those with a lower mean platelet volume (Bs[SE] = − 0.75[0.23] and −0.47[0.20], t[p] = −3.24[.002] and − 2.35[.024], η^2^ = .21 and .12, respectively). Bootstrap procedure confirmed the observed MPV effect with 95% confidence (data not shown).

#### Platelet number & Mean platelet volume

Moderation analysis showed that platelet count and mean platelet volume interacts in their effects on the between-subject difference in mean lnHF-HRV level (PLT_*_MPV interaction) and the profile of reHF-HRV changes (Posture_*_PLT_*_MPV interaction) (Table 3). Bootstrap procedure confirmed the observed interaction effects with 95% confidence (data not shown). The Johnson–Neyman technique with the use of bootstrap confidence intervals detected that mean platelet volume and mean lnHF-HRV level had a significant negative relationship only when the platelet count was higher than 223, and the platelet count and mean lnHF-HRV level had a significant negative relationship only when the mean platelet volume was higher than 9.1. Mean platelet volume and reHF-HRV change (increase) from standing to lying2 (i.e., recovery) had a significant negative relationship only when the platelet count was higher than 246, and the platelet count and reHF-HRV change had a significant positive relationship only when the mean platelet volume was lower than 8.1. No evidence of significant interaction effects of platelet number and mean platelet volume was found for the reHF-HRV change (decrease) from lying1 (baseline) to standing (i.e., for adaptation).

#### Effects of the Sport factor on haemostatic and cardiovascular variables

Significant Sport factor simple effects were obtained for IBI and MPV, and Posture_*_Sport interactions were obtained for IBI, lnHF-HRV, and reHF-HRV (Table 4). Figure 3 presents the latter Sport effect on a profile of between-posture CV changes. Non-athletes showed a flatter profile of CV changes in response to posture changes compared to athletes. Post-hoc analysis found that parameter estimates were significantly lower in non−athletes compared to athletes in general, and particularly during both the lying1 (baseline) and lying2 (recovery) positions for IBI (Bs[SE] = −0.08[0.03], −0.13[0.04], and −0.14[0.04], t[p] = −2.42 [.018], −3.25[.002], and −3.42[.001], η^2^ = .07, .12, and .13, respectively) and during both the lying1 (baseline) and lying2 (recovery) positions for lnHF−HRV (Bs[SE] = −0.90[0.28] and −0.54[0.25], t[p] = −3.26[.002] and −2.15[.035], η^2^ = .12 and .06, respectively). Parameter estimates of reHF-HRV during the lying1 (baseline) position were significantly lower, but during standing were higher in non-athletes compared to athletes (Bs[SE] = −466.0[208.9] and 536.1[199.8], t[p] = −2.23[.029] and 2.68[.009], η^2^ = .06 and .09, respectively). Mean platelet volume was higher in non-athletes compared to athletes (B[SE] = 0.90[0.35], t[p] = 2.56[.015], η^2^ = .14). Bootstrap procedure confirmed the observed Sport factor effects with 95% confidence (data not shown). Since the number of female athletes was only 2 out of 19, the GLM and post-hoc regression analyses with the bootstrapping procedure were redone in males alone and validated the findings (data not shown).

Bootstrapping procedure used here as a robust alternative to inference based on parametric assumptions, confirmed the findings obtained by parametric analyses and additionally detected that the Sport factor changed individual mean and reactivity HF-HRV scores through a mediation mechanism associated with the mean platelet volume. Sport activity decreases mean platelet volume, which in turn increases mean lnHF-HRV during lying (baseline and recovery) positions (Bs[SE] = −0.26[0.18] and −0.20[0.17], 95% CIs = −0.82 - −0.03 and −0.73 - −0.01, respectively), and decreases reHF-HRV during standing (B[SE] = 162.5[130.8], 95% CI = 6.40 - 570.50). Though mean platelet volume and platelet number were significantly negatively correlated (r = −.51, p = .001), the same analyses did not show evidence for platelet number as an outcome of the Sport factor effect nor as a single mediator of the Sport factor effect on HF-HRV measures when compared to mean platelet volume. Since these measures shared a common variance a two-mediator serial model with mean platelet volume and platelet number as mediating variables associated with a common latent (haemostasis) mechanism was tested and confirmed. The serial chain from mean platelet volume to platelet number transmitted the Sport factor effect onto absolute lnHF-HRV value during standing (Sport factor - > MPV- > PLT -> standing lnHF-HRV; B[SE] = 0.22[0.14], 95% CI = 0.01 - 0.60). Sport activity decreases mean platelet volume, which in turn increases platelet number with a decreased mean lnHF-HRV during standing position as outcome.

#### Effects of Gene polymorphism on haemostatic and cardiovascular variables

In the present sample, the *Factor 2* and *GP3a* genes were not polymorphic (Table 2), and no evidence was found for effects related to a Factor 5 gene polymorphism.

#### Factor 12

Significant simple effects of *Factor 12* gene polymorphism were obtained for IBI and lnHF-HRV, and a Posture_*_Factor 12 interaction effect was obtained for lnHF-HRV (Table 4). Post-hoc analysis found similar effects for *CC* and *CT* genotype carriers and, therefore, parameter estimates and Fig. 4A represent simple and interaction effects with the *Factor 12* gene after regrouping to *C*- vs. non-*C*-allele carriers. Non-*C*-allele carriers had significantly lower IBI and lnHF-HRV in general (Bs[SE] = −0.13[0.04] and −0.95[0.28], t[p] = −2.91 [.005] and −3.42[.001], η^2^ = .11 and .15, respectively), and particularly during both the lying1 (baseline) and lying2 (recovery) positions for IBI (Bs[SE] = −0.16[0.05] and −0.16[0.05], t[p] = −3.15[.002] and −2.99[.004], η^2^ = 13 and .12, respectively) and during the lying (baseline) position for lnHF-HRV (B[SE] = −1.67[0.36], t[p] = −4.68[.000], η^2^ = .25). Bootstrap procedure confirmed the observed *Factor 12* effects with 95% confidence (data not shown).

#### MTHFR

Significant simple effect of *MTHFR* gene polymorphism and Posture_*_MTHFR interaction effect were obtained for reHF-HRV (Table 4). Post-hoc analysis found similar effects for *CC* and *CT* allele carriers, therefore, parameter estimates and Fig. 4B represent effects with the *MTHFR* gene after regrouping to *C*- vs. non-*C*-allele carriers. Non-*C*-allele carriers had significantly higher reHF-HRV during lying1 (baseline) and lower reHF-HRV during standing positions (Bs[SE] = 1847.4[397.2] and −1794.2[382.8], t[p] = 4.65[.000] and −4.69[.000], η^2^ = .25 and .25, respectively). Bootstrap procedure confirmed the observed *MTHFR* effects with 80% confidence (data not shown).

#### PAI-1

A significant effect of *PAI-1* gene polymorphism was obtained for PLT (Table 4) with an increase in platelet number from non-*5G* to *5G* allele carriers (Fig. 5A). Bootstrap procedure confirmed the observed *PAI-1* effect with 95% confidence (data not shown). Mediation analysis by bootstrapping procedure detected that a *5G* allele polymorphism of the *PAI-1* gene indirectly increased lnHF-HRV power fluctuation through the mechanism of increasing the platelet number, which was mainly apparent in the increased mean lnHF-HRV during both lying (baseline and recovery) positions (Bs[SE] = 0.27[0.17] and 0.19[0.13], 95% CIs = 0.04-0.79 and 0.04-0.73, respectively).

#### Gene-gene and gene-environment interactions

Several significant effects of gene-gene interaction (Factors 11_*_12) for mean lnHF-HRV level, gene-environment interaction (Sport_*_MTHFR and Sport_*_PAI-1) for mean reHF-HRV and IBI, respectively, and gene-environment interactions (Sport_*_MTHFR_*_Posture and Sport_*_PAI-1_*_Posture) for between-posture IBI, lnHF-HRV, and reHF-HRV changes were obtained (Table 4). Although a bootstrap procedure confirmed the observed interaction effects with 95% confidence (data not shown), these interactions should be evaluated with caution as preliminary due to the small sample size. The *TT* genotype of *Factor 12* compared to *CC* and *CT* genotypes of *Factor 12* significantly decreased mean lnHF-HRV level in only the presence of the *CC* and *CT* genotypes (N = 5 with *Factor 12 TT* vs. N = 52 with *Factor 12 CC* and *CT*) (B[SE] = −1.41[0.31], t[p] = −4.62[.000], η^2^ = .29), but not in the presence of the *TT* genotype of *Factor 11* (N = 2 with *Factor 12 TT* vs. N = 9 with *Factor 12 CC* and *CT*) (B[SE] = 0.22[0.49], t[p] = 0.46[.659], η^2^ = .03) (Fig. 5B). The most pronounced gene-environment effects with increased effects of size were detected for reHF-HRV. The Sport factor had an increased effect on reHF-HRV fluctuating abilities (higher lying1 and lower standing scores) in non-*C* allele carriers of the *MTHFR* gene and *5G*-allele carriers of the *PAI-1* gene (Fig. 6). The GLM analyses of these gene-environment interaction effects with the bootstrapping procedure were also redone in males alone and validated the findings (data not shown).

## Discussion

The study confirmed the hypothesis of an association between the haemostatic factors assessed by the standard complete blood count procedure and cardiovascular fluctuation processes associated with parasympathetic nervous activity (vagus tone and reactivity). Individuals with a lower platelet number or a higher mean platelet volume, which predisposes them to increased bleeding, show a flatter profile of changes of the high frequency component of heart rate variability, either due to low vagus tone or flatter vagus reactivity and recovery during posture changes. These haemostatic factors interact in their associations with the mean and recovery of vagus tone, which suggests a compensatory feedback between these two haemostatic mechanisms in the regulation of parasympathetic nervous activity. Mechanisms associated with mean platelet volume regulate vagal tone in parallel with vagal recovery (both improving with a decrease in volume) when the platelet count is higher than a specific cut-off point (>246). The mechanism regulating platelet count also regulates vagal tone (improving with a decrease in platelet count) separately from vagal recovery (improving with an increase in platelet count) when the mean platelet volume is higher (>9.1) or lower (<8.1) than a specific cut-off point, respectively. These two haemostatic mechanisms seem *to compensate* for each other as a protection against extensive inhibition of vagal tone (i.e., *vagal tone being extensively decreased*) if one of these mechanisms becomes too active (i.e., associated with either a high mean platelet volume or a high platelet count). Vagal recovery seems be regulated differently and the systems *cooperate* in its regulation to prevent this recovery process from becoming too extensive (i.e., *extensive vagal rebound or overshoot after suppression*).

Environmental factors such as sport activity indirectly increase individual vagus tone during lying (rest) and its reactivity (inhibition) in response to standing through regulation of a haemostatic mechanism that decreases the mean platelet volume. Despite the athletes sample size was small, the assumption of the effects and the path was validated through a robust bootstrap procedure. The study also detected evidence that some genetic factors associated with haemostatic mechanisms determine predisposition to low or high vagus tone during lying (rest) and its reactivity (inhibition) in response to standing either directly (*Factor 12* and *MTHFR* gene polymorphisms; the robustness of the latter gene effect was increased in interaction with the Sport activity factor, see below) or indirectly through a platelet count regulation mechanism (*PAI-1* gene polymorphism). Although a group of athletes showed a significant difference in *Factor 5 Leiden* polymorphism compared to non-athletes, it was not associated with between-group differences in vagus activity and measures of the platelet index.

Polymorphism of the *Factor 12* gene was found to determine predisposition to a higher vagus tone in *C*-allele carriers (showing higher plasma Factor XII level and activity^38^), but *MTHFR* gene polymorphism determined predisposition to a higher vagal reactivity (vagus activation at rest and inhibition in response to orthostasis) in non-*C*-allele carriers (predisposing to higher coagulation reactivity^39^). *PAI-1* gene polymorphism determined predisposition to a higher vagus tone in *5G* allele carriers through the increase of the number of platelets. The *PAI-1* gene (*-675 4G/5G*) polymorphism influences the plasma levels of PAI-1, which is considered a regulator of fibrinolysis; *5G/5G* carriers demonstrated a lower level of PAI-1, greater postoperative bleeding and blood loss compared to other variants^40^,^41^. Thus, increased vagus tone, along with platelet count in *5G* allele carriers, may be considered a compensation or resilience mechanism, i.e., increased vagus pro-coagulation activity in these subjects compensates for their predisposition to extensive bleeding.

The gene-environment interaction analysis conducted in this study should be considered preliminary due to the small sample size. However bootstrap confidence intervals were sufficiently robust to validate their presentation and discussion. Sport activity and polymorphism of the *Factor 12* gene were found to have independent effects on vagus tone that suggest the probability of an indirect modification of this gene effect by regular physical activity. However, sport activity directly modifies other genetic effects on vagus reactivity by increasing it in non-*C* allele carriers of the *MTHFR* gene and in *5G*-allele carriers of the *PAI-1* gene, most likely through epigenetic mechanisms, which may heighten the pro-coagulation bias determined by hyperhomocysteinemia in the former group of subjects^39^, but compensate for the coagulation deficit in the latter group of subjects^40^,^41^. An additional gene-gene interaction effect was also detected. The *Factor 12 TT* genotype, which is associated with a Factor XII deficit^38^, demonstrated its inhibitory effect on vagus tone only in the presence of *Factor 11 CC* and *CT* genotypes. The *Factor 11 TT* genotype seems have a protective effect on the pro-coagulation role of vagus tone by encoding high levels of Factor XI (another component of the intrinsic coagulation pathway^42^) in the presence of the impairing effect associated with Factor XII (activating Factor XI).

A challenge for researchers in the post-genomic era is not only to identify the genetic polymorphisms increasing the risk of diseases, but to also determine epigenetic factors and physiological conditioning mechanisms that can counteract the risk genotype by developing a resilient phenotype^1^,^43^. The complexity of the genotype–phenotype relationship of most physiological conditions requires a reliable biomarker of specific physiological resilience with clear mechanisms or paths underlying its phenotypic construction to develop respective therapeutic interventions. This study confirmed the assumption that the high frequency band of heart rate variability was a probable biomarker of such resilience with respect to haemostatic processes. Vagus activity, as a mechanism to determine the power of this heart rate fluctuation, is considered a factor that couples cardiovascular regulation with acquired (mean platelet volume) and innate (coagulation factors XI, XII, PAI-1 [via platelet count regulation], and MTHFR) haemostatic mechanisms, suggesting its prophylactic regulation for promoting advanced protection of an individual against haemorrhage in a variety of combat, occupational, and accidental traumas and injuries, especially in individuals with coagulation deficits. Findings of the present study (e.g., with respect to modifying the generalized effects of *PAI-1* and *MTHFR* gene polymorphisms) may also be important for determining resilience mechanisms against mental health problems like mood disorders and schizophrenia, which severity and treatment outcome were found to be associated with mutations in some coagulation factor genes^44^,^45^,^46^,^47^.

The main limitation of this pilot study is concerned with the small sample size selected for this research. However, several points have been considered for reducing the likelihood of Type I (“false-positive”) and the Type II (“false-negative”) errors in our inferences. In this study more conservative two-tail tests with an alpha of 0.05 were taken while correct directions of most effects are predicted. For example, regular physical exercises (sport factor) have an extremely large effect on vagus tone and reactivity, assessed by HF-HRV. This is reliably defined in previous studies^6^,^7^,^11^,^15^. A sample of athletes was selected in the present study to increase the effect size of the between-subject difference in vagal tone and reactivity. A reasonable sample size of athletes was calculated to detect significance of the sport effect on vagal tone. An obtained direction and size of the effect in the present study are comparable with those from previous studies^48^.

The sample size to detect an association between genetic polymorphisms and vagus activity was chosen to be comparable with the magnitude of sport effect on vagus activity, thus having similar practical importance. In our present study larger group sample sizes would be relevant in the case of gene polymorphisms whose effects do not show evidence of practical importance or statistical significance to avoid type II (“false-negative”) errors in the future. Predicted directions of the relationships between platelet index (platelet count and mean platelet volume) and vagus activity measures^28^,^29^,^37^ and between polymorphisms of coagulation genes and vagus activity measures are also confirmed in present study. Higher platelet number, lower mean platelet volume and genetic polymorphisms (e.g., in *C*-allele carriers of the *Factor 12* gene^38^ and non-*C*-allele carriers of the *MTHFR* gene^39^) that are associated with higher pro-coagulation effects are also associated with higher vagus activity. In the case of *PAI-1* polymorphism (anti-fibrinolytic factor), the correspondence between two indicators of high pro-coagulation activity (high platelet number and high power of HF-HRV) in *5G/5G* carriers of the *PAI-1* gene (having decreased suppression of fibrinolysis^40^,^41^) permits considering the pattern as a part of a physiological mechanism that protect those with this genotype against extensive bleeding.

These results confirm that the present sample size has enough power to demonstrate the predicted vagus-related effects with respect to genetic, haemostatic and physical factors, thereby detecting the mediation relationships between them, in order to support the main hypothesis of their common mechanism. However, gene-gene and gene-environment interaction analyses should be evaluated with caution and repeated in a larger sample size to confirm null results with respect to other genes. Therefore further validation of these findings in a larger population involving more haemostatic factors is required.

## Methods

### Ethical information and participants

This research was performed in accordance with relevant guidelines and regulations, adhered to ethical research standards set by the latest revision of the Declaration of Helsinki, was approved by the Institutional Review Board of the Kazan State University, and informed consent was obtained from all subjects. Data were collected initially during annual health examinations (conducted over 3 different months in the outpatient clinic #4, Kazan) from 19 professional athletes (players and coaches; 2 females; all Caucasians) of the University basketball teams who were recruited by letters to their coaches (mean [range], age 22.5 [17–61]; body mass index 21.6 [15.6–32.5]). Sixty one non-athlete participants (students and faculty staff; 38 females; all Caucasians) were recruited by flyers on University bulletin boards during the same period of time as the annual health examinations (mean [range], age 28.6 [17–66]; body mass index 21.8 [14.9–34.8]).

The effective sample size for testing the main objective of this pilot study was calculated by the power analysis of within- and between-subject effects of physical activity and platelet indices on HF-HRV by using effect size data (η^2^ = .17 and Cohen’s d = 0.53 for exercise, R^2^ = 0.64 for MPV) of previous studies^28^,^48^ and respective total sample sizes = 12 and 10 were found effective for alpha = 0.05 and power = 0.95. However, we increased the total sample size to 80 (40 men and 40 women) to be confident in obtaining sufficient samples with genetic mutations for testing the additional hypothesis of innate mechanisms of these relationships with respect to SNP polymorphisms in genes of some coagulation factors. A sample size with sufficient statistical power is critical to the success of genetic association studies to detect causal effects of genes on physiological and biochemical measures and the effective sample size was computed using Quanto (a program for calculating required sample size for genetic studies) for genetic model with independent subject design and continuous outcomes^49^. Total sample sizes = 40 and 65 were found effective for alpha = 0.05 and power = 0.80 and 0.95 respectively with effect size η^2^ = 0.18 obtained from previous studies for gene-treatment effect on HF-HRV changes^31^. Allele frequencies for rare alleles of the selected factors were found to be from 1.2%–5.1% of 20210A *F2* and 1691A *F5* alleles to 10.5–49.5% of 46T *F12*, 22771T *F11*, 5G *PAI-1*, 196C *GP3a*, and 677T *MTHFR* alleles in European populations^38^,^50^,^51^,^52^,^53^,^54^,^55^.

The participants were qualified for the study after a careful medical record review to determine if inclusion and exclusion criteria were met. Subjects were excluded if they had a history of chronic cardiovascular, pulmonary, renal, liver, or haematological disease, intracranial haemorrhage and haemorrhagic stroke, gastrointestinal or urogenital bleeding, recent major surgery or trauma, a gastric or duodenal ulcer in the past 6 months, elevated levels of aspartate or alanine transaminase (twice above upper-normal range) in the past month, the use of a long-acting non-steroidal anti-inflammatory drug, pregnancy or lactation, or a malignancy.

### Protocol

The study was conducted in the morning after an overnight (at least 8-h) fast during the same week as the annual medical examinations. Subjects were instructed not to consume any alcohol or high-fat meals on the day before the study. On arrival at the lab, participants signed a consent form indicating that the study would consist of (i) measuring height and body weight, causal systolic and diastolic blood pressure and heart rate; (ii) attachment of a device to record respiration parameters; (iii) attachment of a device to record an electrocardiogram (ECG) in response to an orthostatic challenge; (iv) psychological testing (not presented here); as well as (v) obtaining venous blood samples for a complete blood count including platelet number (PLT) and average platelet size (mean platelet volume; MPV), and for identifying SNPs in 7 haemostatic genes, which were of major interest in the study. The procedures were conducted by qualified staff nurses of the clinic in different isolated rooms specifically equipped for each respective procedure. Blood samples were always obtained at the end of the experiment.

### Physiological Measures

Two hundred cycles of a standard limb 3-lead electrocardiogram (ECG) were recorded with a 500 Hz sampling rate in subjects in each of the following positions: lying (first), standing, and lying (second). Measurements were recorded with a “Valenta+” (ECG+respiration) monitoring system (Neo Company, Russia) consisting of a PBS-05 3-lead ECG machine, an MN-02-05.1.1 acquisition system, and a Valenta 1.4 program. The processes that were conducted by the same program included processing ECGs for uneven RR interval series (ms), treating for artefacts, calculating the heart rate (HR; estimated as RR or interbeat interval [IBI] Mode, Mo, s), 10 Hz resampling to the IBI series, detrending, tapering, Fourier transformation and spectral-power value integration in three bands of HR variability (HRV; ms^2^) of which only high frequency (HF-HRV, 0.15–0.40 Hz) was considered as a measure of parasympathetic (PNS) influences on the heart in this study. Because the distribution of the HRV measures was skewed (exponentially distributed), reactivity- and natural log(ln)-transformed HF-HRV indices, as respective relative changes about individual means representing vagus reactivity (reHF-HRV) and absolute values representing vagus tone (lnHF-HRV), were used in statistical analyses^56^. The Valenta 1.4 program also provided a calculation of respiration parameters (respiration rate, vital capacity, forced vital capacity, forced expiratory volume at the 1^st^ s, Tiffeneau Pinelli Index, forced expiratory flow at 25–75%, maximal voluntary ventilation and tidal volume in BTPS expressions) by acquiring the signal from the spirometer. Between-subject variations in the respiration parameters were used to evaluate their possible confounding effects with respect to HRV.

### Blood samples, complete blood count, genotyping procedures and analysis

Blood samples (5 ml) for complete blood count (CBC) and genotyping procedures were obtained from the median cubital vein using vacutainer tubes with K_3_EDTA. For the CBC, all blood samples were analysed within 4 hours of collection. Platelet number (PLT) and mean platelet volume (MPV) values were obtained using the Coulter AcT 5-part differential (5 diff) autoloader (AL) haematology analyser from Beckman Coulter (Fullerton, CA, USA).

Total genomic DNA was isolated from peripheral blood samples using a ‘DNA-Express-Blood’ Kit (Lytech, Russia) according to the manufacturer’s protocol. SNP genotyping of *F2 G20210A* (*rs1799963*), *F5 G1691A* (*rs6025*), *F11 C22771T* (*rs2289252*), *F12 C46T* (*rs1801020*), *PAI-1 4G/5G* (*rs1799768*), *GP3a T196C* (*rs5918*), and *MTHFR C677T* (*rs1801133*)^39^,^54^,^57^,^58^,^59^,^60^,^61^ was performed using a TaqMan probe-based assay (SibDNA, Russia) and a CFX96 Thermal Cycler (Bio-Rad Laboratories, USA). Each sample was processed in duplicate for each genotype analysis. The end-point readings were analysed according to the manufacturer’s instructions.

Of the original sample of 80 subjects, 15 athletes and 55 non-athletes provided DNA and were included in the model analysing genetic effects. Other subjects did not provide DNA due to a variety of reasons (for example, ethical issues concerning a genetic study or genotyping failure).

### Statistical Analysis

Descriptive and inferential analyses were performed with SPSS (SPSS Science, Chicago, IL) software using General Linear Models by the Type III method (GLM), SPSS built-in bootstrapping option for computing confidence intervals for regression estimates in GLM, and the SPSS macro command set to evaluate the significance of moderation and mediation effects^62^. Values of p < .05 were regarded as statistically significant. All parameter estimates are expressed as non-standardized (B) regression coefficients and their standard errors (SE) in the text, and as means and their standard errors in the figures. Where necessary, a partial η^2^ was reported as a measure of strength of associations (effect size). Sex, as categorical, and age (its natural log(ln)-transformed value), as quantitative, variables were included in all models to adjust for factors. Body mass index (BMI) was not included in the analyses because it did not show any significant effect.

**Figure 1:**
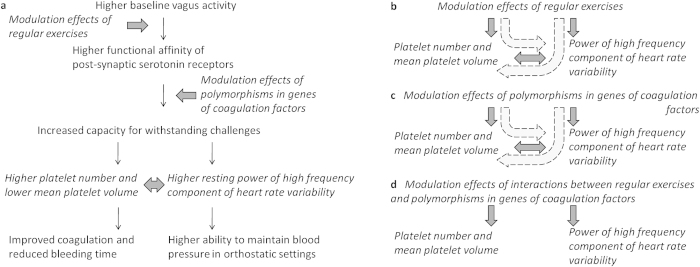
Hypothetical physiological resilience framework (**a**) and schemes of planned statistical analyses (**b**,**c**,**d**). Elements in a regular font depict some hypothetical pathways. Elements in an italic font highlight factors and variables used in general linear (regression and variance) analyses to explore some of these hypothetical mechanisms and possible regulatory effects on them in the present study. The same elements were assessed with confirmation bootstrapping procedures. Two-side bold arrow is related to regression analysis of the main hypothesis. One-side bold arrows are related to the analyses of variance of three other hypotheses. Dashed bold arrows show mediation analyses of indirect effects between variables.

**Figure 2:**
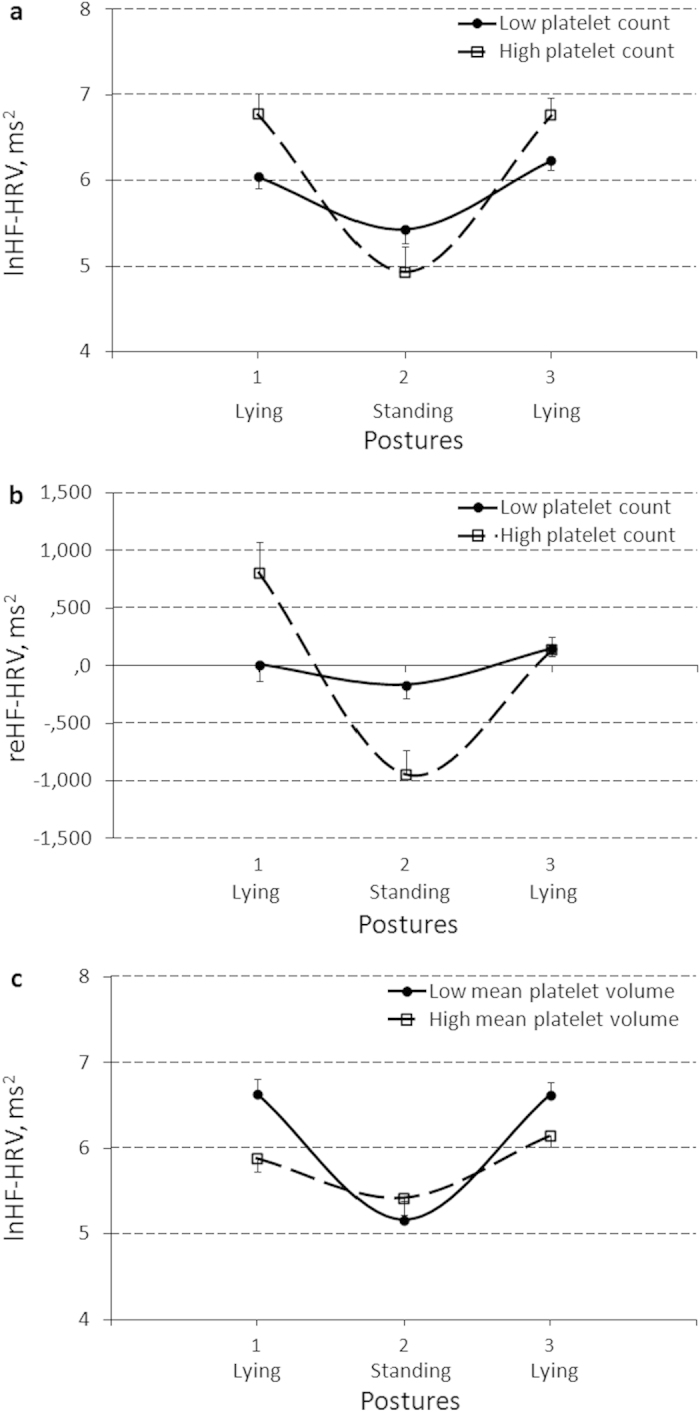
Effect of platelet count (with a score of 242 as the cut-off point) on the profile of between-posture changes of a natural log(ln)-transformed (lnHF-HRV) (**a**), and a reactivity-transformed (reHF-HRV) (**b**) power of a high frequency component of heart rate variability (HF-HRV), and effect of mean platelet volume (with a score of 8.6 as the cut-off point) on the profile of between-posture lnHF-HRV changes (**с**). Results are expressed as means and their standard errors.

**Figure 3:**
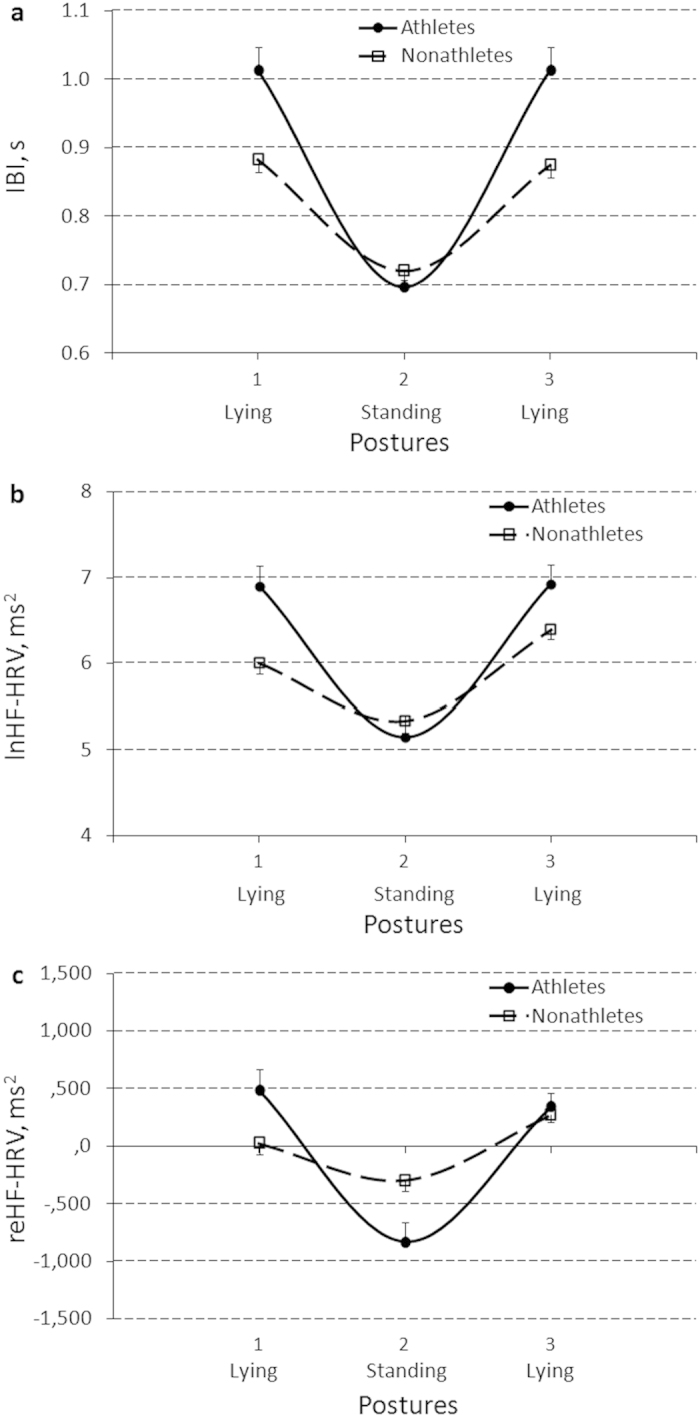
Sport (athletes vs non-athletes) effect on the profile of between-posture changes of interbeat intervals (IBI) (**a**), a natural log(ln)-transformed (lnHF-HRV) (**b**), and a reactivity-transformed (reHF-HRV) (**c**) power of a high frequency component of heart rate variability (HF-HRV). Results are expressed as means and their standard errors.

**Figure 4:**
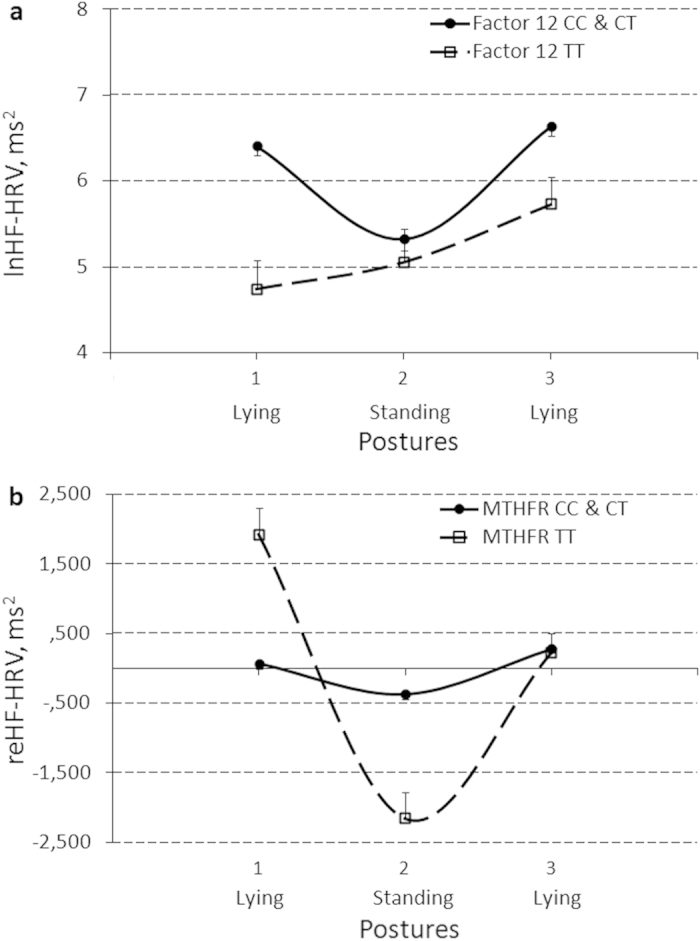
Effects of polymorphism of the *Factor12 gene* (*CC* and *CT* genotype carriers vs. *TT* genotype carriers) on between-posture changes of a natural log(ln)-transformed power of a high frequency component of heart rate variability (lnHF-HRV) (**a**) and polymorphism of the *MTHFR* gene (*CC* and *CT* genotype carriers vs. *TT* genotype carriers) on between-posture changes of a reactivity-transformed power of a high frequency component of heart rate variability (reHF-HRV) (**b**). Results are expressed as means and their standard errors.

**Figure 5:**
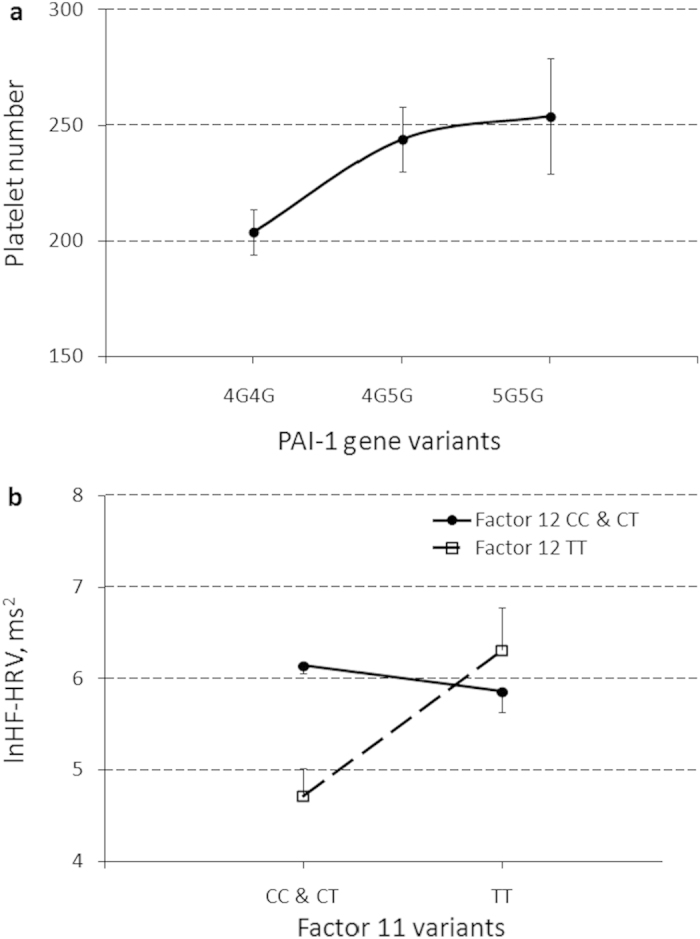
Simple effect of polymorphism of the *PAI-1* gene (*4G4G, 4G5G*, and *5G5G* variants) on platelet count (**a**) and the interaction effect of *Factor 12* * *Factor 11* gene (*CC* and *CT* genotype carriers vs. *TT* genotype carriers) on natural log(ln)-transformed values of the mean power of a high frequency component of heart rate variability (lnHF-HRV) (**b**). Results are expressed as means and their standard errors.

**Figure 6:**
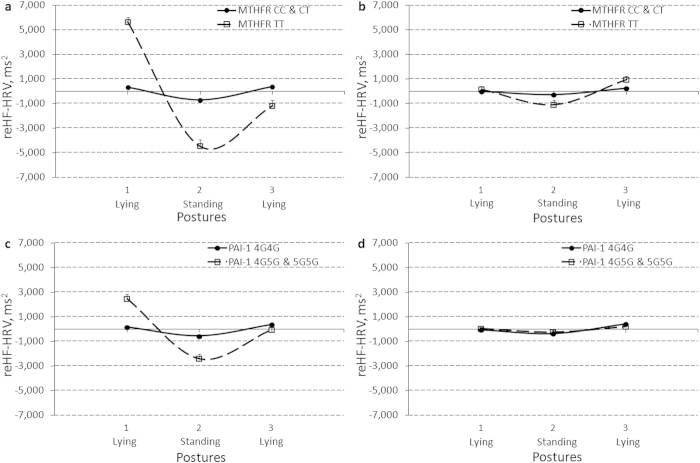
Gene-environment interaction effects on between-posture changes of a reactivity-transformed power of a high frequency component of heart rate variability (reHF-HRV) present in athletes (**a**) and non-athletes (**b**) with *CC* and *CT* genotype carriers vs. *TT* genotype carriers of the *MTHFR* gene, and in athletes (**c**) and non-athletes (**d**) with the *4G4G* vs. *4G5G* and *5G5G* genotype carriers of the *PAI-1* gene. Results are expressed as means and their standard errors.

To confirm the main hypothesis that vagus activity was a generalized protective factor (the general resilience hypothesis), the first group of GLM analyses was conducted to test interaction effects between a within-subject factor (Posture: lying1, standing, lying2) of repeated IBI and HRV measures and between-subject measures of haemorrhages (separately PLT or MPV) treated as continuous independent variables on IBI and HF-HRV measures treated as continuous dependent variables (Fig. 1).

The GLM Repeated Measures procedure provided multivariate analyses of interaction effects of this within-subject factor and haemostatic measures on absolute and relative CV (IBI, lnHF-HRV, reHF-HRV) changes as profile analyses (PLT_*_Posture and MPV_*_Posture). These profile analyses tested the ‘parallelism’ null hypothesis^63^, which asked whether CV changes between postures showed the same pattern (i.e., similar profile) with respect to individual differences in the haemostatic variables. The multivariate approach to repeated measures does not require the compound symmetry and sphericity assumptions. All multivariate F values were obtained by the Pillai’s Trace statistic, which is equivalent to the partial η^2^ measure of effect size.

A second group of GLM analyses was conducted to evaluate three other hypotheses; i.e., that the vagus-related and haemostatic variances could be explained by including: (i) an acquired mechanism (an acquired resilience hypothesis) determined by a conditioning process that is different in athletes and non-athletes and treated as a categorical ‘Sport’ factor (e.g., Sport_*_Posture) (Fig. 1b); (ii) innate mechanisms (an innate resilience hypothesis) determined by polymorphisms of some genes related to haemostatic traits (coagulation factors II, V, XI, XII, PAI-1, GPIIIa, and MTHFR; e.g., MTHFR_*_Posture) treated as categorical independent variables (e.g., with respect to the *MTHFR C677T* genotype as *CC, CT*, or *TT* variant carriers) (Fig. 1c); and (iii) gene-gene and gene-environment interactions (e.g., Factors 11_*_12 and Sport_*_MTHFR_*_Posture) (Fig. 1d). Analyses of the gene-gene and gene-environment interaction effects were conducted and presented as preliminary to provide the effect size for conducting power analysis in future studies of the topic. In addition, significant relationships were evaluated for best-fit cut-off points of respective PLTs and MPVs for presenting these relationships in figures and their possible practical implication.

A third group of mediation and moderation analyses was conducted to evaluate the path and interactions of different resilience mechanisms. All mediation effects were evaluated for significance by the bootstrapping procedure included in the SPSS macro command set. The Johnson–Neyman technique included in the same set was used to detect regions of significant relationships in the cases of significant moderation effects. Degrees of freedom could vary between some analyses due to missing genotypes in some subjects. Compliance with the Hardy–Weinberg equilibrium and the distribution of tested genotypes between groups (athletes and non-athletes) were assessed using the Chi-square and the Freeman-Halton extension of Fisher’s Exact test. Multiple inferences (comparisons) were controlled against Type I errors by cross-validation of observations utilizing the application of different analytic techniques (i.e., parametric and non-parametric profile, bootstrapping mediation, and Johnson–Neyman moderation analyses).

In this study, a bootstrapping procedure (i.e., a procedure with a number of resamples with replacement) was additionally used to validate the findings of regression analyses with uncertain stability (e.g., due to small sample size) by generating 95% confidence intervals for regression estimates in GLM and mediation analyses. Bootstrapping is often used as a robust nonparametric alternative to statistical inference based on parametric assumptions (such as normally distributed errors) and allows assigning measures of accuracy defined in confidence intervals when those assumptions and the stability of the results are in doubt. It can provide more accurate inferences when the data are not well behaved or when the sample size is small^64^. Even when the sample size is as small as 15, the empirical level of the bootstrap test is consistently close to the nominal level^65^. Moreover, the robustness of some conclusions was further increased by applying the bias-corrected and accelerated bootstrap method to the analysis of more complex, indirect or mediating, relationships between grouping factors and dependent measures^62^.

## Additional Information

**How to cite this article**: Davydov, D. M. *et al.* Resilience to orthostasis and haemorrhage: A pilot study of common genetic and conditioning mechanisms. *Sci. Rep.* **5**, 10703; doi: 10.1038/srep10703 (2015).

## Figures and Tables

**Table 1:** Demographic and heart rate characteristics of the samples^1^.

**Groups:**	**Athletes**	**Non-athletes**
***Characteristics***	**Mean (SD)**	**Mean (SD)**
Sex, M/F***	17/2	23/38
Age (year)	22.8 (11.9)	26.4 (14.2)
BMI	22.0 (3.2)	21.8 (3.7)
Caucasian (%)	100	100
		
Lying 1		
HF-HRV** (ms^2^)	1685.7 (2210.8)	686.6 (759.7)
IBI*** (s)	1.022 (.164)	0.881 (.134)
		
Standing		
HF-HRV (ms^2^)	422.1 (595.8)	343.4 (442.4)
IBI (s)	0.706 (.081)	0.717 (.117)
		
Lying 2		
HF-HRV** (ms^2^)	1677.6 (1108.0)	892.4 (879.0)
IBI*** (s)	1.024 (.180)	0.872 (.134)
PLT	220.9 (38.2)	218.2 (46.1)
MPV*	8.0 (0.9)	8.7 (0.7)

^1^means and comparisons (Chi-square test, Mann-Whitney test, and one-way analysis of variance) presented for raw (not transformed and not adjusted) data.

^*^- p < .05.

^**^- p < .005.

^***^- p < .001.

Abbreviations: BMI, body mass index; M, male; F, female; PLT, platelet count; MPV, mean platelet volume; HF-HRV - a power of a high frequency band of heart rate variability; IBI – interbeat intervals.

**Table 2:** Genotype frequencies of studied loci in athletes and non-athletes^a^.

**Genetic loci**	**Groups**		**Athletes**	**Non-athletes**
			**% (N)**	**% (N)**
Factor 2 (prothrombin; Factor II) gene G20210A polymorphism (rs1799963)				
G/G			100 (10)	100 (33)
G/A			0 (0)	0 (0)
A/A			0 (0)	0 (0)
			
Factor 5 (proaccelerin; Factor V) gene G1691A polymorphism (rs6025)*				
G/G			86 (12)	100 (50)
G/A			14 (2)	0 (0)
A/A			0 (0)	0 (0)
			
Factor 11 (plasma thromboplastin antecedent; Factor XI) gene C22771T polymorphism (rs2289252)				
C/C			47 (7)	35 (19)
C/T			40 (6)	46 (25)
T/T			13 (2)	19 (10)
			
Factor 12 (Hageman factor; Factor XII) gene C46T polymorphism (rs1801020)				
C/C			53 (8)	55 (30)
C/T			47 (7)	33 (18)
T/T			0 (0)	12 (7)
			
PAI-1 (plasminogen activator inhibitor-1) gene -675 4G/5G polymorphism (rs1799768)				
4G/4G			77 (10)	44 (23)
4G/5G			23 (3)	40 (21)
5G/5G			0 (0)	16 (8)
			
GP3a (glycoprotein IIIa) gene T196C polymorphism (rs5918)				
T/T			100 (15)	100 (52)
T/C			0 (0)	0 (0)
C/C			0 (0)	0 (0)
			
MTHFR (methylenetetrahydrofolate reductase) gene C677T polymorphism (rs1801133)				
C/C			47 (7)	58 (32)
C/T			47 (7)	38 (21)
T/T			6 (1)	4 (2)

^a^Numbers of individuals varied between different loci due to missing genotypes in some subjects.

^*^Fisher’s Exact p < .05 between the groups.

**Table 3:** Effect sizes (Pillai’s Trace or η^2^) of simple and interaction effects of between-subject differences in platelet number (PLT), mean platelet volume (MPV) and within-subject posture changes (Posture) on interbeat intervals (IBI) and a power of a high frequency band of heart rate variability (HF-HRV) (Univariate and Multivariate Analyses).

**Independent Variables**	**Dependent Variables**			
	**df**	**IBI**	**lnHF-HRV**	**reHF-HRV**
Model 1				
PLT	1	0	0	0.03
Posture	2	0.03	0	0.05
PLT_*_Posture	2	0.09	0.20*	0.14*
				
Model 2				
MPV	1	0.03	0.07	0
Posture	2	0.24**	0.26**	0.27**
MPV_*_Posture	2	0.11	0.14*	0.11
				
Model 3				
PLT	1	0.01	0.14*	0.01
MPV	1	0	0.1	0
Posture	2	0	0	0.1
PLT_*_MPV	1	0.01	0.15*	0.01
PLT_*_MPV_*_Posture	2	0.03	0.02	0.20*

*−p− < 0.05; **−p < 0.005.

All data are adjusted for age and sex.

re- prefix of reactivity transformation of raw data; ln- prefix of natural log transformation of raw data.

**Table 4:** Effect size (Pillai’s Trace or η^2^) of between-subject differences in the Sport factor (athletes vs. non-athletes) and Gene polymorphism^a^ on platelet number (PLT), mean platelet volume (MPV) and in interaction with within-subject posture changes (Posture) on interbeat intervals (IBI) and a power of a high frequency band of heart rate variability (HF-HRV) (Univariate and Multivariate Analyses).

**Independent Variables**	**Dependent variables**					
	**df**	**IBI**	**lnHF-HRV**	**reHF-HRV**	**PLT**	**MPV**
Model 1						
Sport	1	0.07*	0.05	0.01	0.01	0.14*
Posture	2	0.09*	0.06	0.14**		
Sport_*_Posture	2	0.27***	0.14**	0.09*		
						
Model 2						
Factor 12	1	0.11**	0.15**	0.01	0.01	0.02
Posture	2	0.08	0.07	0.15*		
Factor 12_*_Posture	2	0.09	0.19***	0.03		
						
Model 3						
MTHFR	1	0	0.04	0.09*	0.06	0.01
Posture	2	0.11*	0.08	0.20***		
MTHFR_*_Posture	2	0.06	0.01	0.28***		
						
Model 4						
PAI-1	1	0.02	0.04	0	0.21*	0.01
Posture	2	0.07	0.05	0.14*		
PAI-1_*_Posture	2	0	0.02	0.06		
						
Model 5						
Sport	1	0.03	0.03	0.38***	0.01	0.12*
MTHFR	1	0	0.04	0.01	0.04	0
Sport_*_MTHFR	1	0	0.01	0.37***	0.01	0.03
Posture	2	0.13*	0.08	0.30***		
Sport_*_Posture	2	0.25***	0.24***	0.72**		
MTHFR_*_Posture	2	0.09*	0.02	0.69***		
Sport_*_MTHFR_*_Posture	2	0.09*	0.11*	0.68***		
						
Model 6						
Sport	1	0.13**	0.06	0.03	0.08	0.09
PAI-1	1	0.02	0	0.01	0.17*	0.02
Sport_*_PAI-1	1	0.08*	0.02	0.02	0	0
Posture	2	0.11*	0.06	0.15*		
Sport_*_Posture	2	0.38***	0.31***	0.34***		
PAI-1_*_Posture	2	0.12*	0.15*	0.32***		
Sport_*_PAI-1_*_Posture	2	0.12*	0.13*	0.30***		
						
Model 7						
Factor 11	1	0.02	0.07*	0.01	0.03	0.04
Factor 12	1	0.07*	0.04	0	0	0.02
Factors 11_*_12	1	0.02	0.14**	0.01	0	0.07
Posture	2	0.08	0.07	0.13*		
Factor 11_*_Posture	2	0.04	0	0.01		
Factor 12_*_Posture	2	0.03	0.15*	0.02		
Factors 11_*_12_*_Posture	2	0.03	0	0		

*−p < 0.05; **−p < 0.005; ***−p < 0.001.

All data are adjusted for age and sex.

re- prefix of reactivity transformation of raw data; ln- prefix of natural log transformation of raw data.

^a^-*C*- vs. non-*C*-allele carrier grouping of *MTHFR C677T*, *F11 C22771T*, and *F12 C46T* variants and *5G*- vs. non-*5G*-allele carrier grouping of *PAI-1 -675 4G/5G* variants were used in these models.
